# Long-Term Follow-Up of Hypoglossia-Hypodactylia Syndrome: A Case Report

**DOI:** 10.7759/cureus.41290

**Published:** 2023-07-02

**Authors:** Hirotsugu Umeda, Mami Shiraishi, Katsuaki Mishima

**Affiliations:** 1 Department of Oral and Maxillofacial Surgery, Yamaguchi University Graduate School of Medicine, Ube, JPN

**Keywords:** hypoglossia-hypodactylia syndrome, hypoplastic mandible, congenital anomaly, micrognathia, hypodactylia, hypoglossia

## Abstract

Hypoglossia-hypodactylia syndrome is an extremely rare congenital anomaly characterized by a hypoplastic mandible, absence of the lower incisors, hypoglossia, and a variable degree of absence of the digits and limbs, with a risk of dysarthria and dysphagia.

We report the articulation function and the swallowing function of a patient with hypoglossia-hypodactylia syndrome who was followed up to eight years old. Our patient did not have feeding and swallowing disturbances. She did not have articulatory disturbance, including /t/ and /r/, of the sound articulated using a proglossis.

In the future, it is necessary to have a plastic operation for abnormal adhesion of the lower lip and mandibular gingiva and depression on the lower lip, and distraction osteogenesis for micrognathia. Also, it will be necessary to continuously monitor for an articulatory disturbance until the child uses more words. Therefore, a long-term intervention with a multidisciplinary approach is necessary.

## Introduction

Hypoglossia-hypodactylia syndrome (Online Mendelian Inheritance in Man (OMIM) #103300) is an extremely rare congenital anomaly [1]. Hypoglossia-hypodactylia syndrome is characterized by a hypoplastic mandible, absence of the lower incisors, hypoglossia, and a variable degree of absence of the digits and limbs [2]. Intelligence is normal [2]. The etiology is not proven, though there are various reports about hypoglossia-hypodactylia syndrome.

Because of hypoglossia, the development of the risk of dysarthria and dysphagia is concerned, but there have been few case reports of the syndrome. Herein, we report on the articulation function and the swallowing function of a patient with hypoglossia-hypodactylia syndrome who was followed up to eight years old.

## Case presentation

**Figure 1:**
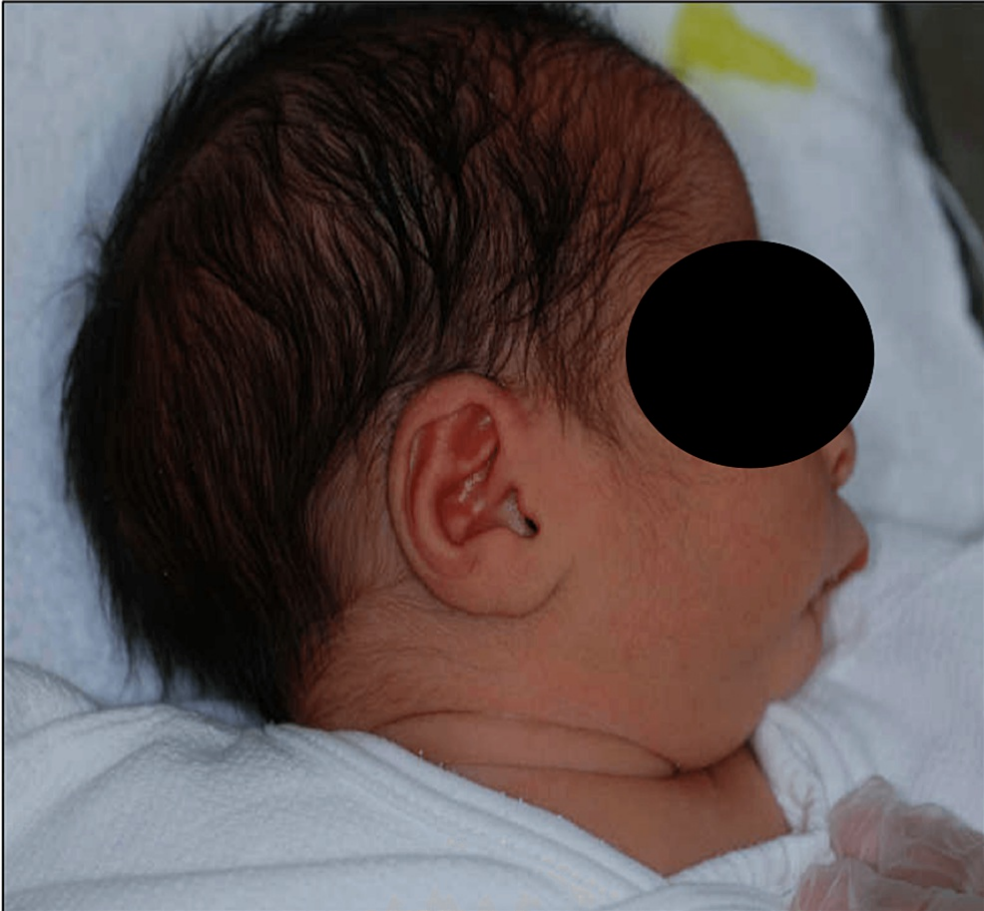
A lateral view of the patient. The patient with micrognathia.

This female infant was born at 38 weeks of gestation by cesarean section. She was born in Yamaguchi, Japan, and was observed at the Department of Oral and Maxillofacial Surgery, Yamaguchi University Hospital. She weighed 2462 g, and her one-minute Apgar score was 8. The infant was the fourth born to a 37-year-old mother and a 40-year-old father. The mother underwent routine prenatal care, including fetal ultrasound. There were no complications during pregnancy. The mother had no history of drug ingestion, abdominal trauma, or X-ray examination during pregnancy. The parents were genetically unrelated. There was no family history of congenital anomalies. The results of chest X-ray, blood test, echocardiography, cranial ultrasound, and abdominal ultrasound examinations were within normal limits, and a chromosomal analysis showed 46,XX normal female karyotype. The patient had micrognathia, but no difficulty breathing (Figure 1).

**Figure 2:**
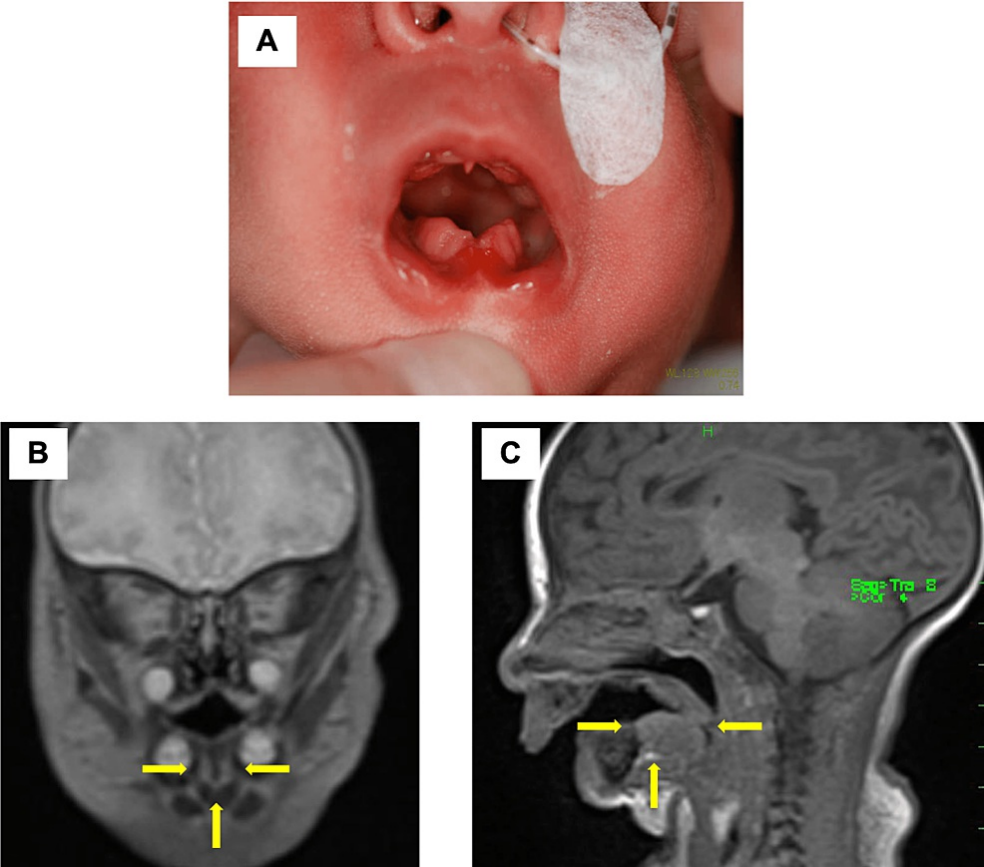
The lower lip and the lower gingiva. A: The lower lip was adhered to the mandibular gingiva. The frenula of the lower lip was fat and short. The alveolar arch of the lower jaw was narrow in V shape. B: MRI (coronal section) of the craniofacial structure. Note the hypoplastic tongue. C: MRI (sagittal section) of the craniofacial structure. Note the hypoplastic tongue.

The lower lip adhered to the mandibular gingiva, and the frenulum of the lower lip was fat and short. The alveolar arch of the lower jaw was narrow and V-shaped, and the tongue was hypoplastic (Figure 2).

**Figure 3:**
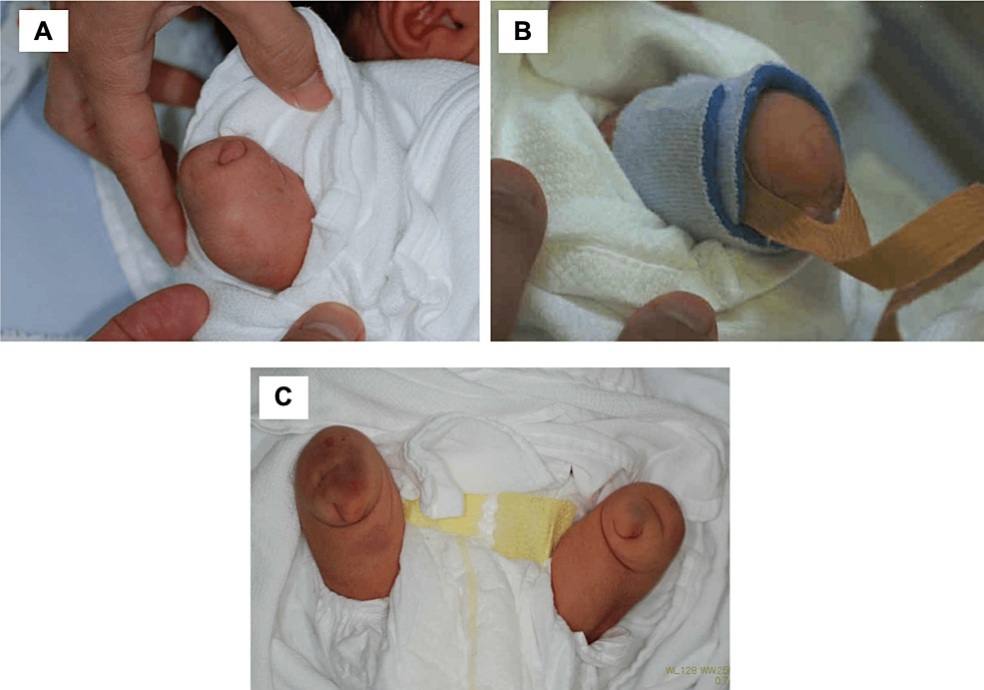
The patient with absence of the digits and limbs. A: The right arm. Note the defect below the elbow. B: The left arm. Note the defect below the elbow. C: Both feet. Note the defect below both articulation genuses.

Other abnormalities were not detected, including cleft palate or high-arched palate. On general physical examination, the infant had a defect distal to both elbows and both knees (Figure 3).

Radiographs showed the defects distal to both humeri and both femora. No other systemic abnormalities were detected.

She started oral feeding with nasal feeding soon after her birth. She was able to orally feed and was only orally fed from her 9th day. She started baby food at six months of age, and she was able to eat normal food at one year and six months of age. The development of eating and swallowing function was favorable. She uttered meaningful words from one year and three months of age, and she began two-word utterances from one year and six months of age. She has been trained by a speech therapist since she was two years of age. At two years and 10 months of age, good mobility of her tongue was able to be confirmed, and articulation of alveolar plosive /t/ and alveolar frill /r/ was enabled. She got normal articulation of all sounds at three years and eight months of age. She was neurodevelopmentally appropriate for her age.

**Figure 4:**
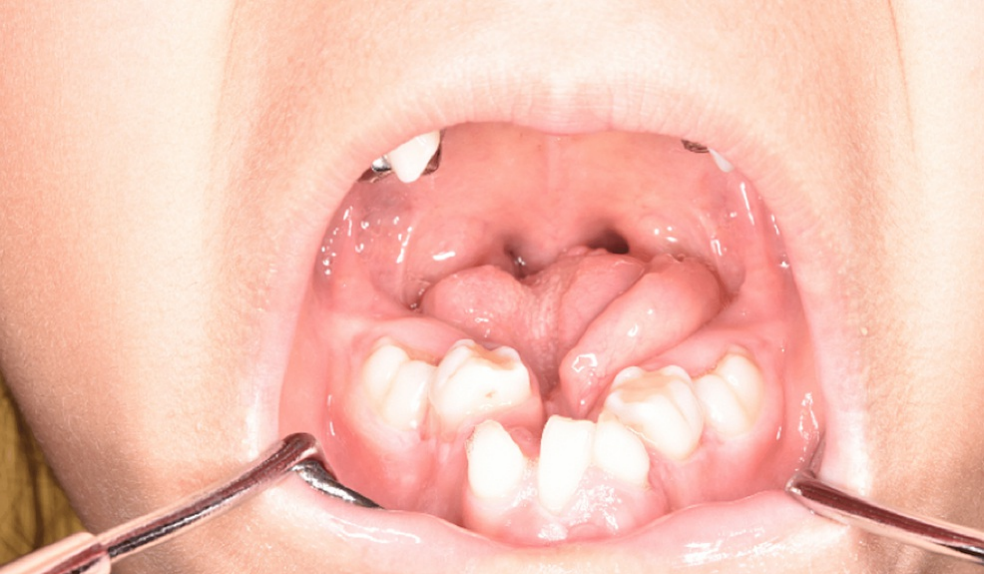
The lower lip and the lower gingiva at seven years and eight months of age. The alveolar arch form did not change and remained narrow and small.

At seven years and eight months of age, the alveolar arch form did not change and remained narrow and small (Figure 4).

With the eruption of the permanent teeth, orthodontics treatment was started. For limb deficiencies, artificial legs and myoelectric prosthetic hands were indicated, and training was continued. She continued to receive speech therapy.

## Discussion

Hypoglossia-hypodactylia syndrome is a difficult disease to treat because the method of forming the tongue to make it functional has not been established. There are many reports of hypoglossia-hypodactylia syndrome [1-5], but the extent of hypoplasia of the tongue and limbs varies. Hypoglossia-hypodactylia syndrome has been classified by Hall as oromandibular and limb hypogenesis syndromes [1]. Oromandibular and limb hypogenesis syndromes are characterized by a hypoplastic mandible, absence of the lower incisors, hypoglossia, and abnormalities of the digits and limbs that range from syndactyly to amelia. It has been classified by Hall into five types with the only necessary criterion being hypoglossia [1]. According to this system, the present patient was classified as a case of type IIA, hypoglossia-hypodactylia. However, it was difficult to distinguish clearly from the clinical findings.

The etiology of hypoglossia-hypodactylia syndrome is unknown. Scott [6] has shown the presence of organized thrombi in the fetal stem vessels on microscopic examination and has suggested that damage to the distal limbs might result from an ischemic or embolic event associated with placental fetal vessel thrombosis. In the present patient, the event that caused deterioration of the intrauterine environment, such as abdominal trauma, during pregnancy was not known. Teratogenic factors in embryonic life have been suggested as among the etiologies of this syndrome [7]. Certain drugs have been known to have been administered to mothers during the critical period of facial and limb development [6], including meclizine hydrochloride [8]. Oulis and Thornton [9] reported that one patient’s mother had regularly undergone chest X-rays during pregnancy. In the present patient, no drug usage or radiation exposure was reported by the parents. Additionally, genetic factors have been reported, but many cases occur sporadically [10]. In the present patient, no genetic factor was reported by the parents. Thus, the etiologies of hypoglossia-hypodactylia syndrome remain unknown.

Micrognathia is one of the features of hypoglossia-hypodactylia syndrome [6]. A hypoplastic mandible with concurrent hypoglossia could be explained by the fact that the mandible originates from the same visceral arch as the tongue [11]. Since micrognathia may cause occlusal abnormalities and sleep apnea, surgical orthodontic treatment is more likely to be required in the future.

Hypoglossia-hypodactylia syndrome may disturb oral functions, and hypoglossia has the potential for causing tongue movement disorders. Tongue movement disorders cause feeding disturbances, swallowing disturbances with a risk of pneumonia, and articulatory disturbances. In a majority of cases, there are no problems with feeding and swallowing [12], and oral ingestion is good. However, sounds articulated using the proglossis, such as /t/ and /r/, may become unclear. Imai et al. [13] reported a patient with hypoglossia-hypodactylia syndrome who was diagnosed with mild dysarthria by a speech therapist. Rasool et al. [12] also reported that the development of speech was retarded and obscured in most cases, but in the present patient, the development of speech and articulation were normal. In this syndrome, it is considered that problems with articulation such as alveolar plosive may occur and that a palatal augmentation prosthesis is indicated in such a case. Unfortunately, there have been no reports on the use of the palatal augmentation prosthesis for this syndrome. In the present patient, dysarthria was not observed. It was speculated that the tip of the tongue probably reached the palate by reducing the oral volume by some method such as raising the floor of the mouth.

In this syndrome, oral feeding is difficult after birth, and nasal tube feeding is provided in some cases, but oral ingestion becomes often possible until the age of one to three years [14]. Ardran et al. [15] evaluated swallowing function by X-ray for this syndrome and reported that the soft tissue of the tonsil pushed the bolus posteriorly and sent it from the pharynx to the esophagus. In the present patient, swallowing and feeding functions were normal, and aspiration pneumonia was not observed. In addition, there was no problem in shifting to baby food or normal food. In this syndrome, dysphagia and eating disorders caused by hypoplasia of the tongue are concerned, but it is considered that swallowing function and eating function may be obtained by compensating for the tissues surrounding the tongue.

There are few reports on the management and therapy of occlusion in this syndrome [13,16,17]. Heggie et al. [17] have reported that distraction osteogenesis to improve the size and shape of the hypoplastic mandible has recently been applied for this syndrome. Imai et al. [13] have reported that a good occlusal relationship was obtained by receiving orthodontic treatment after expanding the dental arch by distraction osteogenesis. In the present patient, a narrow and V-shaped mandibular arch was left, and she is receiving orthodontic treatment to expand the dental arch.

## Conclusions

We followed hypoglossia-hypodactylia syndrome in a patient with hypoplasia of the tongue and limb anomalies until she was eight years old. In the future, plastic surgery will be required to improve aesthetics and dysfunction due to abnormal adhesion between the lower lip and the lower gingiva and depression of the lower lip. Also, as the child uses more words, it will be necessary to monitor for an articulatory disturbance. Therefore, a long-term intervention with a multidisciplinary approach is necessary.
